# Protective Effects of an Octapeptide Identified from Riceberry™ (*Oryza sativa*) Protein Hydrolysate on Oxidative and Endoplasmic Reticulum (ER) Stress in L929 Cells

**DOI:** 10.3390/foods13152467

**Published:** 2024-08-05

**Authors:** Sucheewin Krobthong, Theeranuch Jaroenchuensiri, Yodying Yingchutrakul, Pichayapa Sukmak, Wonnop Visessanguan, Pawin Pongkorpsakol, Tatpong Tulyananda, Chanat Aonbangkhen

**Affiliations:** 1 Center of Excellence in Natural Products Chemistry (CENP), Department of Chemistry, Faculty of Science, Chulalongkorn University, Bangkok 10330, Thailand; 2 National Center for Genetic Engineering and Biotechnology, National Science and Technology Development Agency (NSTDA), Pathum Thani 12120, Thailand; 3 Princess Srisavangavadhana College of Medicine, Chulabhorn Royal Academy, Bangkok 10210, Thailand; 4 Plant Biology & Astrobotany Laboratory, School of Bioinnovation and Bio-Based Product Intelligence, Faculty of Science, Mahidol University, Salaya Campus, Nakhon Pathom 73170, Thailand; 5 Center of Excellence on Petrochemical and Materials Technology, Chulalongkorn University, Bangkok 10330, Thailand

**Keywords:** Riceberry™, rice, oxidative stress, antioxidant peptides, VPAGVAHW, LC-MS/MS

## Abstract

Reactive oxygen species (ROS) play a critical role in oxidative stress and cellular damage, underscoring the importance of identifying potent antioxidants. This research focuses on the antioxidant capabilities of Riceberry™-derived peptides and their protective effects against oxidative and endoplasmic reticulum (ER) stress in L929 cells. By simulating human digestion, Riceberry™ protein hydrolysate was generated, from which antioxidant peptides were isolated using OFFGEL electrophoresis and LC-MS/MS. Notably, an octapeptide (VPAGVAHW) from the hydrolysate demonstrated significant antioxidant activity, particularly against oxidative stress induced by iodoacetic acid (IAA) or hydrogen peroxide (H_2_O_2_) and ER stress caused by tunicamycin (TM) in L929 cells. This peptide’s effectiveness was evident in its dose-dependent ability to enhance cell viability and mitigate stress effects, although its efficiency varied with the stress inducer. Our study suggests that Riceberry™-derived peptides could serve as a promising natural antioxidant with potential benefits for health promotion and applications in the food industry, offering an environmentally friendly alternative to synthetic antioxidants.

## 1. Introduction

Free radicals, comprising a spectrum of unstable and highly reactive chemical entities, are ubiquitous in biological systems. They originate from both endogenous metabolic processes and exposure to exogenous factors, such as ultraviolet rays, air pollutants, industrial chemicals, and xenobiotics. In their optimal state within normal cells, free radicals play pivotal roles in various cellular functions, contributing to the maintenance of cellular health and functionality [1]. However, an imbalance, particularly an elevation in free radical levels, can have detrimental effects on living organisms at the cellular level, leading to oxidative stress and potential damage to vital macromolecules, including proteins, DNA, and lipids [2]. A major class of these free radicals is reactive oxygen species (ROS), encompassing diverse molecules such as peroxides, hydrogen peroxide, hydroxyl radicals, superoxide anion radicals, and singlet oxygen [3]. ROS are primarily generated in several cellular compartments, notably the endoplasmic reticulum (ER) and mitochondria. Excessive ROS production can lead to cellular oxidative stress, causing damage to cellular components, disrupting redox homeostasis, and leading to physiological dysfunctions [4]. The ER, a central organelle in the cell, is responsible for the quality control of protein folding. It facilitates the folding and modification of proteins and manages the degradation of misfolded proteins. These processes are tightly regulated by the redox state within the ER microenvironment, influenced by factors such as thiol–disulfide exchange reactions, glutathione ratios, and calcium homeostasis [5,6]. Impaired ER function is linked to an imbalance in redox homeostasis and excess ROS production, which are conditions associated with a range of diseases, including cataracts, cardiovascular diseases, diabetes mellitus, neurodegenerative diseases, respiratory diseases, rheumatoid arthritis, and cancers. As ROS are a major concern for human health issues, the discovery of potential antioxidants is needed. Because of the harmful effects of ROS and the need to maintain cellular redox balance, the exploration and discovery of effective antioxidants have become a crucial area of research. Peptides, especially those sourced from edible materials, have emerged as promising candidates in the search for natural antioxidants. The enzymatic hydrolysis of food materials produces smaller peptides with various functional properties, including antibacterial, anti-angiotensin-converting enzyme (ACE), anti-tumor, anti-lipid accumulation, and antioxidant activities [7,8,9]. The antioxidant capabilities of these peptides are largely dependent on their amino acid composition and sequence arrangement. Research has uncovered various peptides with antioxidant properties from different food sources, including nonapeptides (VDLPTCKGF) from Lingzhi [10], heptapeptides (TVRPPQR) from green algae [11], and three antioxidant peptides (DSTRTQ, DVYSF, and ESKPV) from cooked eggs [12]. Therefore, finding new antioxidant peptides derived from various foods has been rapidly ongoing.

Riceberry™ rice, a novel and exotic source of natural antioxidants has recently garnered significant attention. As a staple food in many parts of the world, Riceberry™ plays a crucial role in human nutrition. Riceberry™ has been protected under plant variety protection laws since 2017, with successful trademark registrations in both Thailand and other countries. Derived from a cross-breed of Hom-Nil rice and Jasmine rice, Riceberry™ is rich in antioxidants, such as 4-hydroxybenzoic acid, chlorogenic acid, ferulic acid, gallic acid, p-coumaric acid, syringic acid, and vanillic acid [13]. In addition, studies have shown that proteins and peptides found in Riceberry™ exhibit notable antioxidant capacities and can counteract cellular oxidative stress [14]. Various endoproteases such as actinase, neutrase, trypsin, and alcalase were used for the digestion of proteins in rice to produce a diverse array of antioxidant peptides, each with unique amino acid compositions and molecular weights [15]. However, there is a lack of research that mimics human digestion processes to demonstrate the potential benefits of protein hydrolysates in human health. Despite this, the abundance and economic viability of Riceberry™ protein hydrolysates make them a promising source for the discovery of novel antioxidant peptides.

The identification of new antioxidant peptides from Riceberry™ requires sophisticated chromatographic techniques. One such technique is OFFGEL electrophoresis, which allows for the fractionation of complex peptide mixtures based on their isoelectric points (pI), facilitating the elimination of interfering substances such as salts, phenolic compounds, and lipids. OFFGEL electrophoresis, a technique based on immobilized pH gradient (IPG) strips, separates peptides in an electric field according to their pI. This approach offers high separation efficiency and allows for the easy recovery of fractionated peptides in a solution [16]. Here, the objective of this study was to identify and investigate the antioxidant properties of peptides derived from Riceberry™ through dual enzymatic digestion using pepsin and trypsin. Peptides isolated from Riceberry™ protein hydrolysate possess significant antioxidant properties, which alleviate oxidative and ER stress in mammalian cells. It was hypothesized that some peptides could enhance cell viability and mitigate the effects of oxidative stress induced by iodoacetic acid (IAA) or hydrogen peroxide (H_2_O_2_) and ER stress in L929 cells induced by tunicamycin (TM) in a dose-dependent manner. L929 cells are part of a mouse fibroblast cell line, which is commonly used for biological studies. Our investigation also included examining cell morphology and the intensity of ER-localized dyes, like lectin-concanavalin A (ConA), to assess the change in the ER. This study not only highlights the potential of specific Riceberry™-derived peptides in mitigating ER stress but also underscores their potential to enhance the economic value of Riceberry™ as a functional food ingredient for health promotion in various food industries.

## 2. Materials and Methods

### 2.1. Materials and Reagents

The chemicals, materials, and equipment used in this work were as follows: Riceberry™ grain was collected in August 2020 and dried under 50 °C in a hot-air incubator for 72 h. We used the reference rice verities obtained from Pathumthani Rice Research Stations (Rice Department, Ministry of Agriculture and Cooperative, Thailand). The materials and equipment used in this analysis included a UV-Vis spectroscopy Synergy H1 microplate reader purchased from BioTek Co. (Winooski, VT, USA). Vivaspin^®^ 20 was purchased from GE Healthcare Co. (Amersham, UK), and Sep-Pak C18 cartridges were obtained from Waters Co. (Milford, MA, USA). L929 cells were purchased from the American Type Culture Collection (ATCC, Manassas, VA, USA). Biochemical reagents, including Dulbecco’s Modified Eagle Medium (DMEM) and Fetal Bovine Serum (FBS), were purchased from Gibco (Grand Island, NY, USA). The penicillin G, dimethyl sulfoxide (DMSO), MTT, LPS, pepsin originated from porcine pancreatic (250 U/mg), 2,2-diphenyl-1-picrylhydrazyl (DPPH), ascorbic acid (As), Gallic acid (GA), 2,2′-azino-bis(3-ethylbenzothiazoline-6-sulfonic acid (ABTS), K_2_S_2_O_8_, FeCl_3_, and Nylon filter membranes (pore size 0.22 μm) were purchased from Sigma Aldrich Co. (St. Louis, MO, USA). Trypsin originated from bovine pancreatic (6000 U/mg) and was purchased from Promega Co. (Madison, WI, USA). All other reagents were purchased from Sigma Aldrich Co. Solvents for LC-MS, including LC-MS waters and acetonitrile (ultra-LC-MS), and were purchased from J.T. Baker (Fisher Scientific, Loughborough, UK).

### 2.2. In Vitro Oral, Gastric and Intestinal Protein Hydrolysate Preparation

The in vitro protein digestion process was adapted from previous reports with minor modifications [7]. Initially, Riceberry™ grains (40 g) were ground and mixed with deionized water in a 1:5 (*w/v*) ratio in an Erlenmeyer flask. This mixture was then autoclaved under standard conditions (121 °C at 103 kPa for 20 min) and subsequently cooled to room temperature. For simulating oral digestion, the autoclaved Riceberry™ was subjected to stirring at 25 rpm in a shaker. A solution containing 50 mL of α-amylase (1 U/mL concentration in a buffer of 50 mM NaCl, 5 mM KCl, and 5 mM CaCl_2_) was then added, and the sample was incubated at 37 °C for 10 min. Following oral digestion, the sample’s pH was adjusted to 1.8 using 6 N HCl, preparing it for gastric digestion. Pepsin was added to achieve a final concentration of 100 U/mL, and this reaction was maintained for 60 min. To transition simulating intestinal digestion, the pH was first adjusted to 5.5 using 2 M NaHCO_3_. Pancreatin (to reach a final concentration of 100 U/mL based on trypsin activity) and bile salts (to a final concentration of 10 mM) were then introduced into the mixture. The pH was further adjusted to 7.2 using 2 M NaHCO_3_, and this stage of digestion proceeded for 6 h. To stop the enzymatic reactions, the solution was heated to 95 °C for 5 min. The hydrolyzed mixture was then mixed with ethanol in a 1:4 (*v/v*) ratio and centrifuged at 12,000× *g* for 30 min at 16 °C to separate large starch particles. Finally, the supernatant was collected and dried under a vacuum using a rotary evaporator.

### 2.3. Protein Hydrolysate Purification and Fractionization

To fractionate peptides based on their isoelectric points using OFFGEL fractionation, the dried pellet of hydrolyzed proteins was first reconstituted in deionized water (DI water). This solution was then filtered through a 0.22 µm nylon membrane and concentrated using a Vivaspin^®^ 20 concentrator with a 10 kDa molecular weight cut-off. The filtrate underwent solid-phase extraction (SPE) using C18 cartridges, which were pre-conditioned with 50 mL of acetonitrile and then equilibrated with 100 mL of water. The hydrolyzed protein solution was loaded onto the prepared SPE cartridges and eluted with 40% acetonitrile in DI water. The eluted fraction was evaporated using a rotary evaporator under vacuum and subsequently reconstituted in DI water. For fractionation by an isoelectric point, an 18 cm-long immobiline drystrip gel was employed as the matrix support to separate the peptides. The OFFGEL device was configured to separate into 18 fractions over a linear pH gradient ranging from 3 to 10. Each well of the strip was equilibrated with 60 μL of a 0.2% IPG buffer (pH 3–10) and incubated for 60 min. Then, 3.6 mg of the sample (resolubilized 60% acetonitrile pellet fraction) was dissolved in DI water to a final volume of 3.6 mL. An aliquot of 200 μL of this sample was loaded into each well, and the wells were covered with mineral oil to prevent evaporation. Fractionation was conducted at a maximum current of 50 μA and a constant temperature of 20 °C until the total voltage reached 32 kVh. Post-focusing, the solutions from each well were collected in individual centrifugal tubes. These peptide fractions were then concentrated by vacuum centrifugation in preparation for antioxidant capacity determination.

### 2.4. DPPH and ABTS Radical Scavenging Assays

Radical scavenging assays were conducted to find the highest antioxidant fractions from OFFGEL fractionation. The evaluation of the relative antioxidant capacity of the fractionated proteins was carried out using two different reagents, including 2,2-diphenyl-1-picrylhydrazyl (DPPH) and 2,2′-azino-bis(3-ethylbenzothiazoline-6-sulfonic acid (ABTS) [17]. Antioxidant capacity was determined at the fixed volume of 20 mL (10% of total reaction volume) of all fractions. For DPPH antioxidant activity, the hydrolysate was mixed with 180 µL of 0.2 mM DPPH in ethanol. The control was composed of 20 μL of ethanol and 180 μL of the 0.2 mM DPPH solution. The absorbance of the mixture was evaluated at 517 nm. For ABTS antioxidant activity, an ABTS radical solution (7 mM ABTS stock solution with 2.45 mM potassium persulfate) was diluted in 5 mM of phosphate buffer saline solution, pH 7.4. The hydrolysate was mixed with a 180 µL ABTS solution. The control was composed of 20 μL of deionized water and 180 μL of the ABTS radical solution. The reaction was incubated at room temperature in the dark for 10 min. The absorbance of the mixture was evaluated at 734 nm. Antioxidant capacity from both the DPPH and ABTS assay was calculated according to the following equation:

(1) $$DPPH\;and\;ABTS\;radical\;scavenging\;activity\;(\%)=\left(1-\frac{Abs(Sample)}{Abs(Control)}\right)\times 100$$

where Abs_sample_ is the absorbance of the DPPH or ABTS solution with the testing sample, and Abs_control_ is the absorbance of DPPH or ABTS containing all reagents except for the testing sample.

The antioxidant capacity determined from both the DPPH and ABTS assays was expressed in terms of the IC_50_ value. The IC_50_ value was calculated by assessing the scavenging activity of various dilutions of each test sample and interpolating the concentration of the peptides at which the percentage of inhibition reached 50%.

### 2.5. De Novo Sequencing for Peptide Identification Using LC-MS/MS 

The antioxidant peptide sequence was identified from the highest antioxidant capacity fractions of OFFGEL fractionation. The highest antioxidant fraction was dried and reconstituted in 0.1% formic acid and transferred to a TruView LCMS vial (Waters, Milford, MA, USA UK). A total of 200 ng of protonated peptides were subjected to LC-MS/MS. Spectrum data were collected in the positive mode on an Orbitrap HF hybrid mass spectrometer combined with an UltiMate 3000 LC system. Briefly, the protonated peptides were first desalted online on a reverse-phase C18 PepMap 100 trapping column before being resolved onto a C18 PepMap 100 capillary column with a 120 min gradient of 0.1% HCO_2_H/H_2_O (mobile phase (MP): A) and 0.1% HCO_2_H/CH_3_CN (MP: B), at a flow rate of 300 nL/min. Peptides were analyzed by applying a data-dependent Top5 acquisition mode, followed by a higher-energy collisional dissociation (HCD) at collision energy = 28. Full scan (MS) mass spectra were acquired from *m/z* 400 to 1600 with an AGC target set at 10^6^ ions and a resolution of 120,000. An MS/MS scan was initiated when the ACG target reached 2 × 10^5^ ions and a resolution of 30,000. The raw mass spectra (.raw file) were processed by the PeakX studio 10.0 program [18]. De novo peptide sequencing was performed with the default parameters of the Orbitrap instrument. Briefly, the LC-MS run was analyzed with no specific digestion enzyme. The mass deviation tolerances for MS and MS/MS were 10 ppm and 0.01 Da, respectively. The HCD-fragmentation series was used to construct peptide sequences [18]. The confidence score was configured at a high level to obtain a small set of peptides with high precision. The acceptable de novo peptide sequences were achieved by filtering the average local confidence (ALC) to ≥95%. 

### 2.6. Investigation of the Cytotoxic Effect of the Synthetic Peptides on L929 Cells

Cell cytotoxicity was evaluated by the MTT assay. L929 cells were seeded in a 96-well plate at a density of 10^5^ cells per well. The cells were then treated with peptides for a duration of 24 h. After the incubation period, the peptides were removed from the wells. To evaluate the cytotoxicity of the peptides at 50 µg/mL, an MTT assay was performed. The negative control (NegCtrl) consisted of wells containing only cells in a culture medium without any peptide treatment. The absorbance of the formazan product, proportional to the number of viable cells, was measured at a wavelength of 570 nm using a microplate reader (PerkinElmer, EnSight Multimode Microplate Reader). The determination of the cytotoxic effects of the peptides on the cells by comparing the absorbance values of the treated cells with those of the NegCtrl, the relative cell viability and potential cytotoxicity of the peptides at 50 µg/mL could be assessed using the average cell viability percentage with standard deviation (S.D.) values. The experiments were conducted in two independent replicates (*n* = 2). Additionally, each independent experiment included three well replications (*n* = 3). 

### 2.7. Validation of Antioxidant Ability by Measuring Intracellular ROS in Oxidative Stress-Induced-L929 Cells

For the evaluation of the protective effects of antioxidative stress peptides in cells, the peptide candidates with the highest antioxidant capacity in vitro were chosen. The peptides were reconstituted in a culture medium to achieve a final concentration of 50 µg/mL, following the methodology outlined in the previous study. The L929 cells were seeded in a 96-well plate, allowing them to attach and grow for 24 h. Subsequently, the peptides were added to the cells and incubated for 24 h. Following the incubation, the peptide was removed, and the cells were subjected to oxidative stress induction using iodoacetic acid (IAA) or hydrogen peroxide (H_2_O_2_) at selected concentrations for a specified duration, 3 h for IAA and 24 h for H_2_O_2_. Cell viability was assessed using the MTT assay, as described above. The control was untreated cells without an oxidative stressor (cells with only a cell culture medium), and the treated cells had only oxidative stressors (no peptide treatment). By comparing the absorbance values of the treated cells with those of the NegCtrl condition, the relative cell viability and potential cytotoxicity of the peptides at 50 µg/mL could be assessed in the average cell viability percentage with S.D. values. The experiments were conducted in two independent replicates (*n* = 2). Additionally, each independent experiment included three well replications (*n* = 3).

### 2.8. The Effect of the Antioxidant Peptide on Tunicamycin (TM)-Induced ER Stress in L929 Cells

The L929 cells were seeded in 8-well chamber slides at a density of approximately 50,000 cells per well. Then, 50 µg/mL of the selected peptide was pre-treated and added to the cells along with tunicamycin (TM) at a concentration of 10 µg/mL for 24 h. To prepare the cells for imaging, the cells were fixed and permeabilized using a solution of 4% paraformaldehyde for 15 min followed by treatment with Triton X-100 for 2 min to ensure proper cell permeabilization. Subsequently, the cells were stained with concanavalin A (ConA)-conjugates with FITC, and the nuclear staining dye DAPI (at a concentration of 1 µg/mL) to visualize specific cellular structures.

Cellular morphology and staining patterns were observed using a confocal fluorescence microscope (ZEISS Axio Observer LSM 980). The negative control consisted of cells treated with a 1% DMSO solution in the culture medium (as TM was dissolved in DMSO). The positive control of TM alone involved cells treated with 10 µg/mL of TM, which was diluted in the culture medium. In our experimental study, we employed the corrected total cell fluorescence (CTCF) method to calculate the fluorescence intensity followed by Equation (2) [19].

(2) $$CTCF\;=\;Integrated\;Density-\left(Area\;of\;selected\;cell\times Mean\;fluorescence\;of\;background\right)$$

where the integrated density was the sum of fluorescence values within the selected cell area, and the area of the selected cell was the total number of pixels within the selected cell area. The mean fluorescence of the background was the average fluorescence intensity of background regions measured in areas devoid of cells.

### 2.9. Statistical Analysis

All the results were performed in three independent experiments in order to determine their reproducibility expressed as the mean ± S.D. Tukey’s test was used to compare the effect of differences between the means of each group by Design-Expert 11.0 (Minneapolis, MN, USA). Analysis of variance was employed to examine the differences amongst treatments at *p*-value < 0.05. 

## 3. Results

### 3.1. Purification and Fractionation of Antioxidant Peptides

**Figure 1:**
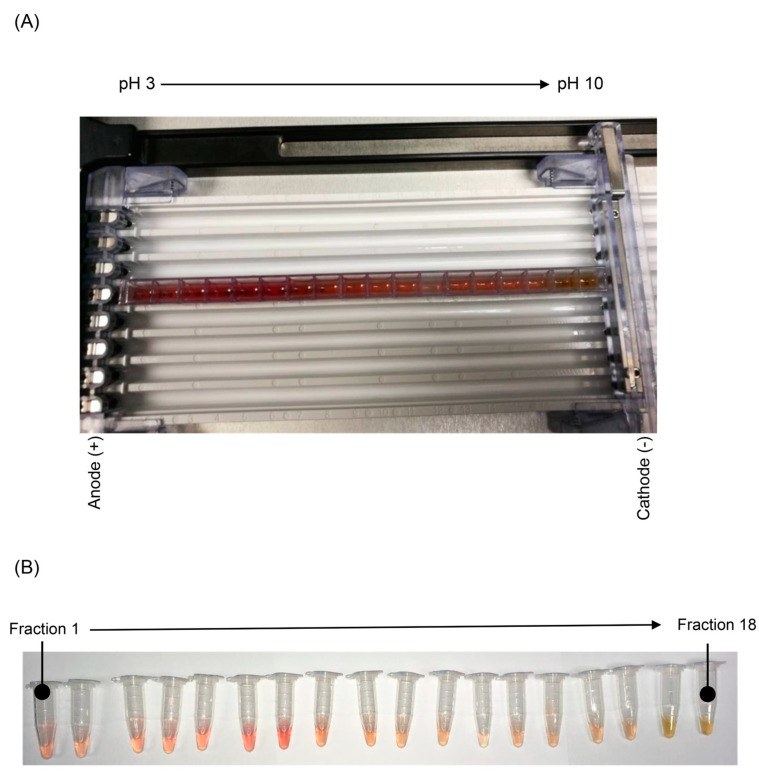
Illustration of peptide fractionation via OFFGEL fractionation. (**A**) The OFFGEL experiments involved the separation of the 40% acetonitrile fraction following 3 h of electro-focusing using an electric field across a pH gradient ranging from 3 to 10. (**B**) The resulting 18 fractions are shown in individual microcentrifuge tubes after separation. Each tube corresponds to a specific pH range, displaying the distribution of peptides visually. The gradient of colors from red to yellow indicates the separation efficiency and the variation in peptide content across different pH values.

By employing OFFGEL fractionation, the protein hydrolysate of Riceberry™ was effectively partitioned into 18 distinct fractions, as illustrated in Figure 1A. The peptides, once fully fractionated, were systematically gathered from their respective wells, as depicted in Figure 1B. A grand total of 18 fractions were collected and subsequently subjected to lyophilization and biochemical assays to determine their antioxidant capabilities in vitro. To assess their potential as antioxidants, we employed the DPPH assay for lipid-soluble radicals and the ABTS assay for water-soluble radicals, enabling us to identify the fractions with the highest antioxidant efficacy.

### 3.2. In Vitro Antioxidant Screening Assays of Fractionated Peptides

Out of the 18 fractions obtained through OFFGEL fractionation, 16 showed radical scavenging activities. The DPPH and ABTS radical scavenging abilities were quantified as IC_50_ values for all these fractions, as detailed in Table 1. As depicted in Table 1, the antioxidant activity showed a gradual increase with the increase in the isoelectric points of peptides, particularly around a pH of 7. Nevertheless, it is noteworthy that as the peptides’ isoelectric points reached approximately 9.2 or higher (Fraction 17–18), the antioxidant activity became undetectable.

Fraction 13 (with a pI range of 7.67–8.06) exhibited the highest antioxidant capacities in both the DPPH and ABTS assays, showing outstanding DPPH and ABTS scavenging activities with IC_50_ values of 12.31 ± 0.08 and 21.69 ± 0.13 µg/mL, respectively. Notably, a statistically significant difference was observed between the DPPH and ABTS activities of fraction 13 and the other fractions (*p*-value < 0.05). Consequently, fraction 13 was chosen for peptide identification via de novo sequencing by LC-MS/MS.

### 3.3. Antioxidant Peptide Candidate Identifications Using De Novo Sequencing

The de novo algorithm was conducted to predict peptide sequences derived from ion peaks, ensuring the absence of any ambiguous sequences. Our dataset comprised 17,521 MS/MS spectra corresponding to peptides with charge states of +2, +3, and +4. The peptide spectrum-matching process yielded 11 peptides, each with a length ranging from 7 to 14 amino acids, averaging at 9.36 residues and possessing molecular weights within the range of 766.38 to 1521.88 Da, as shown in Table 2.

These peptides were randomly fragmented into the daughter peptide ions spectrum (MS/MS) (Supplementary Data S1). The assignment of peptide sequences to the MS/MS spectrum relied on comparing their mass values with those of successive peptide b- and y-ion series. While individual y- or b-ions alone may not provide the complete peptide sequence, the integration of both y-ion and b-ion series enables the construction of the entire peptide sequence without any ambiguous amino acid residues, eliminating the need for residue predictions. Notably, this approach achieved a high level of accuracy, with more than 95% of the b- and y-ions present in the spectrum being successfully identified. This comprehensive identification contributed to the generation of a full MS/MS spectrum, further reinforcing the reliability of peptide sequencing.

### 3.4. Evaluation of Cell Cytotoxicity from Synthetic Peptides in L929 Cells

To further assess the antioxidant capacity of peptide sequences obtained from OFFGEL fractions and LC-MS/MS, the peptides in fraction No. 13 were re-synthesized using the Solid Phase Peptide Synthesis (SPPS) approach. Quality control measures, including evaluations of peptide purity and mass accuracy, were rigorously applied. The purity levels of these peptides varied between 92% and 97%. Furthermore, the molecular masses of the peptides were accurately determined by analyzing the peptide ions in the LC-MS spectra, exhibiting a deviation range from 0.016% to 0.083%.

**Figure 2:**
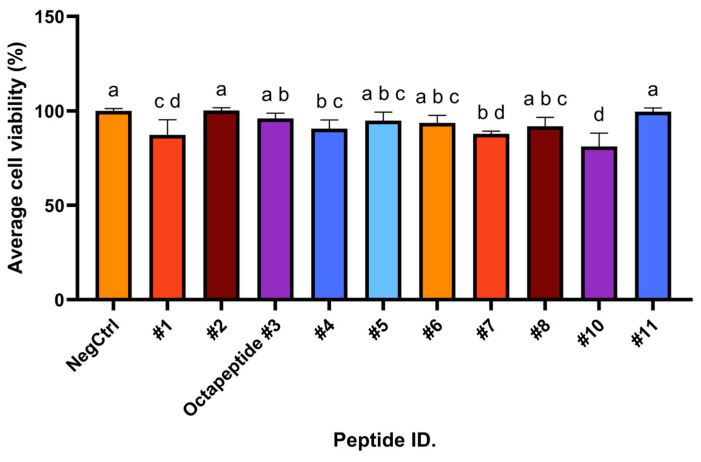
Effect of synthetic peptides on cell viability in L929 cells. The synthetic peptides at 50 µg/mL were tested by incubating cells with the peptides for 24 h, and the cytotoxicity was determined by the MTT assay. The lowercase letters above the bar graph indicates the significance of differences in cell viability between treatment groups. The symbol # followed by a number indicates the name of each peptide.

First, to underscore the importance of safety in peptide applications, we conducted a focused assessment of the potential adverse effects of these synthetic peptides on a normal cell line, L929, of mouse fibroblast cells, which are commonly used for biological studies. The investigation was performed at a concentration of 50 µg/µL, as illustrated in Figure 2. 

Different peptides exhibited different levels of cell viability. Groups sharing the same letter are not significantly different from each other at the 0.05 confidential level. Specifically, peptides #1 (PEHYLDHFKL) and #10 (TLKYPLE) showed the most significant reduction in cell viability (*p*-value < 0.001), represented by the letter d, indicating that they were significantly different from the negative control (NegCtrl), and several other peptide groups. Peptides #2 (FYDPKTPFF), NegCtrl, and #11 (DVVHSHASN) showed the highest cell viability when grouped under letter a. Octapeptide #3 (VPAGVAHW) is in the intermediate group with letters a and b, while peptides #4 (LKELGDKVPAPVKE), #5 (LDDPAKKLVFGGSA), #6 (PASVAHW), and #8 (AKLPPGSD) were in groups a, b, c. Peptides #4 (LKELGDKVPAPVKE) and #7 (LDDPAKKLVF) fell into groups b and d, suggesting intermediate cytotoxicity effects. However, solubility issues with peptide #9 (LLKLPTL) in the culture medium were encountered, leading to its exclusion from the experiments. 

### 3.5. Validating the Antioxidant Capacity of Synthetic Peptides

The antioxidant capacity of these synthetic peptides was evaluated using two distinct methods: the DPPH and ABTS assays. Our findings revealed that individual synthetic peptides exhibited lower antioxidant capacities compared to the combined peptides in fraction 13. All of the peptides, except octapeptide #3, demonstrated IC_50_ values exceeding 50 µg/mL in both DPPH and ABTS assays. Notably, octapeptide #3 was the only one that stood out with IC_50_ values of 40.51 ± 1.54 µg/mL for DPPH and 28.32 ± 1.09 µg/mL for ABTS. Due to its high antioxidant capacity, as measured by the DPPH and ABTS assays, octapeptide #3 was selected for further investigation to assess its rescue effect on cell viability in oxidative stress-induced L929 cells.

### 3.6. Validating the Rescue Effect of Antioxidant Peptides in L929 Cells under Iodoacetic Acid (IAA) and H_2_O_2_-Induced Oxidative Stress

**Figure 3:**
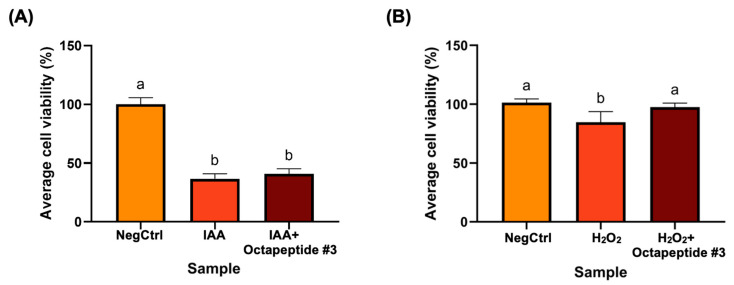
The impact of octapeptide #3 on L929 cells under oxidative stress induced by iodoacetic acid (IAA) and H_2_O_2_. (**A**) Illustrates the protective effect of octapeptide #3 on IAA-induced L929 cells, and (**B**) shows the protective effect of octapeptide #3 on H_2_O_2_-induced L929 cells. The percentage of cell viability is represented as the mean ± S.D. The lowercase letters above the bar graph indicates the significance of differences in cell viability between treatment groups.

The effect of octapeptide #3 on oxidative stress in L929 cells, induced by iodoacetic acid (IAA) and hydrogen peroxide (H_2_O_2_), was investigated. The viability of cells under oxidative stress was assessed after treatment with the peptide at a concentration of 50 µg/µL, as depicted in Figure 3.

In Figure 3A, Tukey’s multiple comparison test reveals that the IAA-treated alone and the IAA with octapeptide #3-treated groups (labeled b) show significantly lower cell viability compared to the vehicle control (NegCtrl labeled a). The negative control had a mean viability of 100%, while IAA reduced it to 36.58%, and octapeptide #3 showed an intermediate protective effect with a mean viability of 40.84%. In Figure 3B, similar results were obtained with the H_2_O_2_ treatment. The vehicle control (NegCtrl labeled a) also showed significantly higher cell viability compared to the H_2_O_2_ treated alone and the H_2_O_2_ with octapeptide #3-treated groups (Figure 3B). The negative control had a mean viability of 101.30%, while H_2_O_2_ reduced it to 84.65%, and octapeptide #3 showed an intermediate protective effect with a mean viability of 97.53%.

With pair-wise comparisons, we observed that octapeptide #3 treatment, prior to IAA stimulation, significantly reduced cell viability compared to the NegCtrl condition (*p*-value < 0.01) (Figure 3A). However, in H_2_O_2_-induced L929 cells, octapeptide #3 did not show a significant decrease in cell viability when compared to the NegCtrl condition (*p*-value = 0.51) (Figure 3B). In experiments using IAA as the stress inducer, octapeptide #3 did not demonstrate a significant difference in cell viability (*p*-value = 0.32) compared to the oxidative stress condition induced by IAA (Figure 3A). Conversely, in experiments employing H_2_O_2_ as the stress inducer, octapeptide #3 showed a significant improvement in cell viability compared to cells treated with H_2_O_2_ alone (*p*-value = 0.005). 

### 3.7. Investigation of the Protective Effect of Octapeptide #3 on Tunicamycin (TM)-Induced ER-Stress in L929 Cells Using Confocal Fluorescence Microscopy

**Figure 4:**
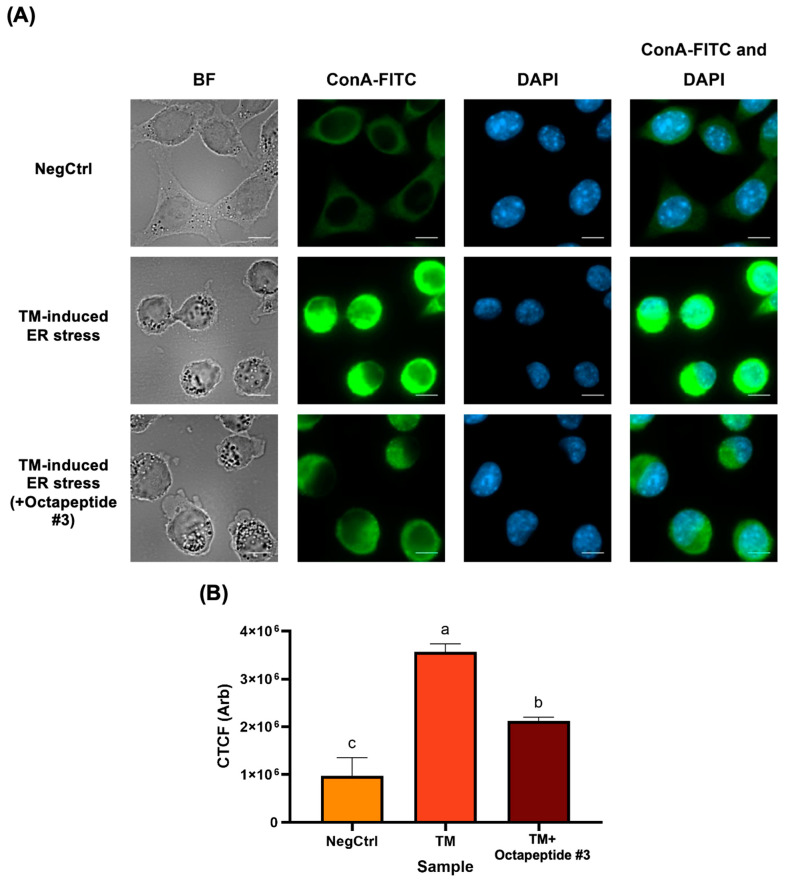
The impact of octapeptide #3 on tunicamycin ™-induced ER stress in L929 cells. (**A**) Cell morphology visualized by confocal fluorescence microscopy. The staining includes ConA-FITC, emitting green fluorescence to highlight ER stress, and DAPI, emitting blue fluorescence to delineate the nuclear regions. ‘BF’ denotes the bright field images. (**B**) A comparative analysis of CTCF values among the NegCtrl, TM treated alone, and TM treated followed by octapeptide #3 treatment groups. The CTCF values are presented as the mean ± S.D. The lowercase letters above the bar graph indicates the significance of differences in cell viability between treatment groups.

The confocal fluorescence microscopy images in Figure 4A illustrate the morphological changes and endoplasmic reticulum (ER) stress levels in L929 cells under different treatment conditions. In the NegCtrl group, the cells exhibited normal morphology with well-defined ER structures and clear, round nuclei, indicating minimal or no stress as a reference for normal healthy cells. In contrast, the tunicamycin (TM)-treated cells showed significant distress, as can be seen with the cell shrinkage, rounding, and intense ConA-FITC staining (ER-specific lectin conjugated with the FITC green-fluorescent dye), indicative of high ER stress levels. The nuclei in these cells appeared condensed or fragmented, suggesting cellular stress and early apoptosis. Notably, cells treated with octapeptide #3 under ER stress conditions maintained a more normal morphology, with less shrinkage and rounding compared to the TM-treated group. ConA-FITC staining is less intense, indicating reduced ER stress and the ER structure appeared more organized. Additionally, the nuclei in octapeptide #3-treated cells were more similar to those in the NegCtrl group, with fewer signs of condensation or fragmentation. Altogether, this suggests that octapeptide #3 exerted a protective effect against TM-induced ER stress in L929 cells.

Comparative analysis of the corrected total cell fluorescence (CTCF) was conducted. Figure 4B presents a comparative analysis of CTCF values among NegCtrl, TM treated alone, and TM with octapeptide #3-treated groups, illustrating the impact of these treatments on ER stress in L929 cells. The NegCtrl group, serving as the baseline, exhibits the lowest CTCF values, indicative of minimal ER stress. In contrast, the TM-treated group showed a significant increase in CTCF values, approximately a 263% increase compared to NegCtrl, highlighting the substantial ER stress induced by TM. This is statistically significant, with a *p*-value < 0.0001. The octapeptide #3-treated group showed an intermediate CTCF value, with an approximately 118% increase compared to NegCtrl, indicating a significant reduction in ER stress compared to the TM-treated group but still higher than the NegCtrl. This difference is statistically significant with a *p*-value of 0.0014. Moreover, the TM group exhibited an approximately 40.64% higher CTCF value compared to the octapeptide #3 group, further confirming the protective effect of octapeptide #3 against TM-induced ER stress, with this difference being statistically significant at a *p*-value = 0.0002. These findings collectively demonstrate that while TM induces high ER stress, octapeptide #3 significantly mitigates this stress, though not completely to the level of the negative control.

## 4. Discussion

In our previous work, we established a correlation between the antioxidant activity of crude hydrolyzed proteins from various food sources and the molecular weight of water-soluble peptides [7,20]. Specifically, we found that peptides with relative molecular weights less than 3 kDa exhibit significant radical-scavenging capabilities [10]. This observation is consistent with findings from other studies on diverse food protein sources such as eggs, milk, and algae, which have also yielded antioxidative peptides with slightly lower molecular weights [11,12,21]. Moreover, the selection of an appropriate molecular weight range is essential to produce a more standardized product, ensuring the desired molecular mass range while eliminating any residual undigested proteins and digestion enzymes. Riceberry™ is known to be a rich source of natural antioxidants, primarily anthocyanins [22], which belong to the phenolic phytochemical subclass. Anthocyanins typically exhibit a red color in acidic conditions. As seen in Figure 1B, the acidic fractions (fractions 1–8; pH~3–6) appear red, likely due to the presence of anthocyanins. The OFFGEL fractionation technique proves highly effective in removing color compounds and obtaining high-purity antioxidant peptides. Furthermore, OFFGEL fractionation allows for the precise separation of peptides based on their pI without the need for additional chromatographic purification steps. This method maintains the peptides in solution, streamlining the process and enhancing the purity of the final product.

The peptide sequences were determined exclusively through the de novo algorithm in conjunction with LC-MS/MS. Our high-resolution MS instrument, featuring a resolution of 120,000 for MS and 30,000 for MS/MS, enabled the confident identification of numerous internal fragments, totaling 17,521 spectra. Additionally, it allowed us to identify more than a single fragmentation ion product per peptide. To validate the accuracy of these peptide sequences established through de novo sequencing, we performed alignment using the BlastP program against non-redundant protein sequences (nr) in the database [23]. An available online database-assisted sequence matching platform for de novo peptide sequencing is more reliable and can be used for this species because a fully decoded genome database is available [24]. Notably, most of the identified peptides originated from the protein digestion of Riceberry™, with 9 out of the 11 peptides found to be in complete alignment with the *Oryza sativa* protein database. This alignment strongly supports the precision of the de novo sequencing algorithm.

Two short novel peptides, LLKLPTL and TLKYPLE, are particularly interesting because they have not been reported as part of any proteins in the *O. sativa* database. Furthermore, there is no prior mention of the relationship between antioxidant activity and these peptides. Our selection of these peptides was based on the high accuracy of their sequences, as indicated by ALC scores exceeding 95%. However, it is noteworthy that these peptides also exhibit certain characteristics in line with previous reports, such as their short peptide chains (less than 25 amino acid residues) [25,26] and the presence of a high composition of hydrophobic amino acids, including phenylalanine, proline, glycine, and alanine, within their sequences [27]. The antioxidant mechanism associated with hydrophobic amino acids involves their ability to act as electron subtractors or proton donors, effectively neutralizing free radical molecules, including hydroxyl, peroxide, and superoxide anion species [28]. Additionally, the prevalence of hydrophobic amino acids enhances the peptides’ affinity for the water–lipid interface, thereby facilitating their access to and removal of free radicals at the interface.

To validate and evaluate the antioxidant ability of these peptides, SPPS was conducted to synthesize the peptides. In general, synthetic peptides of >80% purity are considered appropriate for biochemical assays [29]. Therefore, we can infer that these peptides were successfully synthesized for cell-based experiments and the antioxidant validation assay by DPPH and ABTS methods. Octapeptide #3 exhibited scavenging capacity measuring via the IC_50_ of DPPH and ABTS lower than 50 µg/mL. Our finding suggests that a single peptide has a lower antioxidant capacity compared with mixture peptides in fraction No.13 from OFFGEL fractionation. In fraction No.13, the diversity of peptides, each with unique amino acid sequences, contributes to a broader range of chemical interactions with free radical molecules. This diversity of the peptides allows for more effective scavenging of different types of free radicals. In contrast, octapeptide #3 has a limited range of chemical interactions due to its specific amino acid sequence (only eight residues), potentially leading to a narrower capacity for antioxidant activity. In addition, the reduced antioxidant capacity of octapeptide #3 compared to a peptide mixture from fraction No.13 may be caused by the lack of synergistic effects, where various peptides may work together to enhance overall efficacy [30]. Peptides with various chemical interactions and synergistic effects may target various oxidative pathways through complementary biochemical cascades, offering a broader range of antioxidant protection in the cells [31]. A limitation of this study is the absence of conditions involving intact digestive enzymes (α-amylase, pepsin, pancreatin, trypsin) during the evaluation of the effects of octapeptide #3 against oxidative and ER stress in cell-based experiments. The OFFGEL fractionator used for fractionation is not suitable for intact enzymes due to its large size and structural complexity, which hinder their mobility through the IPG strip’s gel matrix, resulting in poor resolution and incomplete separation. Furthermore, large proteins are more prone to denaturation during the fractionation process, compromising their structural integrity. To focus on digestible peptides and small compounds (<3 kDa), we employed a molecular weight cut-off device that effectively filtered out these intact proteins. However, incorporating the effects of intact digestive enzymes would require an alternative experimental approach that does not use the molecular weight cut-off device, potentially introducing additional variables and complicating the interpretation of the results. Consequently, this study does not account for the direct impact of intact digestive enzymes on octapeptide #3, which is a limitation that should be addressed in future research.

Antioxidant peptides derived from various natural sources have shown promising potential in protecting cells from oxidative damage. These peptides, typically produced through the enzymatic hydrolysis of proteins, have been extensively studied for their ability to scavenge free radicals and mitigate oxidative stress. For instance, peptides from the protein hydrolysate of *Mytilus edulis* demonstrated protective effects on human umbilical vein endothelial cells against oxidative stress via the downregulation of p53 and caspase-3 genes [32]. Antioxidant peptides from the muscle protein hydrolysate of monkfish (*Lophius litulon*), such as the sequences YWDAW, EDIVCW, and MEPVW, were found to protect HepG2 cells from H_2_O_2_-induced oxidative damage by enhancing the cellular antioxidant defense system [33]. Furthermore, peptides derived from tuna byproducts, particularly from milts, like the sequences WGESF, IKSW, YSHM, and WSPGF, exhibit antioxidant functions that protect HUVECs from the oxidative stress caused by H_2_O_2_ [34]. However, our work showed potential differences from previous reports in several aspects. Firstly, Riceberry™ is a unique and underexplored source of antioxidant peptides. The peptides identified in our study, especially VPAGVAHW, have not been reported in other sources, highlighting the novelty of our findings. Secondly, our work demonstrates the dual functionality of the identified peptides in protecting against both oxidative and ER stress, which is a relatively unexplored area in the context of antioxidant peptides. This dual protective mechanism underscores the potential application of Riceberry™-derived peptides in health promotion and disease prevention, particularly in conditions where oxidative and ER stress play a crucial role.

We realized the differences between in vitro assays and cell-based assays, prompting us to conduct cell-based experiments for more realistic mechanisms in the cells. We, therefore, focused on the impact of octapeptide #3 on cell cytotoxicity and the rescue effect in stressed cells, acknowledging the importance of toxicological information for using peptides as functional food ingredients (Supplementary Figure S1). At a concentration of 50 µg/mL, none of the synthetic peptides showed toxicity. In particular, octapeptide #3, with the highest antioxidant capacity, increased cell viability in H_2_O_2_-induced oxidative stress in L929 cells at this concentration. However, its impact on IAA-induced oxidative stress in L929 cells was not significant. To investigate deeper, we doubled the octapeptide’s concentration in subsequent experiments, concentrating on IAA-induced oxidative stress in L929 cells. Intriguingly, at 100 µg/mL, octapeptide #3 considerably enhanced cell viability compared to cells treated solely with IAA. This led to an increase in cell viability to 72.26%, or a 1.77-fold increase, compared to the group treated with 50 µg/mL of the octapeptide. These results suggest that octapeptide #3 provides a dose-dependent protective effect against oxidative stress. Nonetheless, the enhanced viability observed in octapeptide #3-treated groups highlights its potential in mitigating the detrimental effects of H_2_O_2_ on cell survival. To evaluate the antioxidant capacity of octapeptide #3 and assess its protective effects against ER stress, the choice of an appropriate cell model is crucial. L929 cells from a fibroblast cell line derived from the subcutaneous connective tissue of a mouse were chosen for this study due to their sensitivity to oxidative stress and their active ER functions. These cells are particularly suitable for studying oxidative stress as they respond well to oxidative stress-inducing agents such as H_2_O_2_ and IAA, allowing for the precise measurement of antioxidant efficacy. 

Octapeptide #3 demonstrated a more pronounced protective effect against H_2_O_2_-induced oxidative stress compared to IAA-induced oxidative stress in L929 cells. It is important to note that H_2_O_2_ and IAA induce oxidative stress through distinct mechanisms. H_2_O_2_ primarily triggers oxidative stress and cellular damage but also acts as a signaling molecule in cell proliferation, differentiation, and apoptosis. This action is moderated by antioxidant systems, including catalase and glutathione peroxidase [35]. In our study, a higher concentration of octapeptide #3 was required to improve cell viability in IAA-induced L929 cells, possibly due to IAA’s involvement in a broader biochemical cascade that disrupts various pathways. This is in contrast to H_2_O_2_, the primary role of which is to generate ROS and induce oxidative stress. Our experiments, conducted with both stress inducers, aimed to investigate the specificity of these stressor molecules. The results suggest that octapeptide #3 is more effective in mitigating the extensive ROS generated by hydrogen peroxide, highlighting their specificity in protecting cells against this type of oxidative stress.

The protective effect of octapeptide #3 on ER stress in L929 was observed. We used TM as an ER stress inducer. TM is a known chemical that is widely used to inhibit glycosylation and disrupt protein folding in ER [36]; this leads to misfolded proteins accumulating in the ER. TM also disrupts calcium homeostasis in the cells, leading to ER stress [37]. We used CTCF to quantify and represent the fluorescence signal of ConA-FITC as an ER-specific agent to measure the level of ER stress. CTCF is a measurement technique used with the ImageJ program to quantify the fluorescence signal in the cell by calculating the intensity of fluorescence for each individual cell and background subtracted. Higher CTCF values indicate a higher intensity of fluorescence within the cell. Octapeptide #3, treated in our study, as shown in Figure 4, demonstrated a protective effect on TM-induced ER stress in L929 cells. In a more detailed examination of the octapeptide #3 sequences, we noted an interesting study that reported an 8-residue peptide (VLATSGPG) from salmon skin (*Salmo Salar*) effectively suppressing ER stress. This peptide inhibits PERK activation through the AKT signaling pathway in HepG2 cells [38]. Notably, there are similarities in molecular weight and sequence between this peptide and those identified in our experiments. Both feature hydrophobic amino acid residues at their N- and C-termini. This similarity has drawn our interest, prompting further investigation into potential targets and molecular mechanisms associated with ER stress regulation in cells.

## 5. Conclusions

This work explored the antioxidant capacity of peptides obtained from Riceberry™ protein hydrolysate. Using OFFGEL electrophoresis and LC-MS/MS, specific antioxidant peptides were isolated and identified. These peptides demonstrated varying degrees of antioxidant activity in vitro, with one particular octapeptide, #3, showing outstanding antioxidant potential with the VPAGVAHW sequence. Further investigations assessed the protective effects of these peptides on L929 cells under oxidative stress induced by iodoacetic acid (IAA) and H_2_O_2_ and endoplasmic reticulum (ER) stress induced by tunicamycin (TM). Octapeptide #3 exhibited a dose-dependent protective effect against oxidative stress, improving cell viability significantly in H_2_O_2_-induced stress. The octapeptide showed a protective effect on ER-stressed cells, suggesting its ability to preserve cellular morphology and reduce the adverse impact of ER stress. Overall, this study underscores the promise of Riceberry™-derived peptides, particularly the identified octapeptide #3, as a potent antioxidant with potential applications in health promotion and as functional food ingredients. 

## Figures and Tables

**Table 1:** The free radical scavenging activity of all fractions in the pH range 3 to 10 obtained from the OFFGEL fractionation experiment.

Fraction	pH Range	IC_50_ (mg/mL)	
		DPPH	ABTS
Fraction 1	3.00–3.39	90.21 ^o^ ± 0.13	66.37 ^p^ ± 0.15
Fraction 2	3.39–3.78	98.42 ^p^ ± 0.22	60.23 ^m^ ± 0.07
Fraction 3	3.78–4.17	89.13 ^n^ ± 0.17	63.21 ^o^ ± 0.19
Fraction 4	4.17–4.56	66.22 ^k^ ± 0.07	55.38 ^k^ ± 0.25
Fraction 5	4.56–4.94	72.52 ^l^ ± 0.16	59.32 ^l^ ± 0.13
Fraction 6	4.94–5.33	80.07 ^m^ ± 0.11	61.81 ^n^ ± 0.12
Fraction 7	5.33–5.72	61.90 ^i^ ± 0.07	43.12 ^h^ ± 0.05
Fraction 8	5.72–6.11	63.81 ^j^ ± 0.19	45.30 ^i^ ± 0.09
Fraction 9	6.11–6.50	55.69 ^g^ ± 0.08	54.73 ^j^ ± 0.17
Fraction 10	6.50–6.89	22.44 ^c^ ± 0.01	29.92 ^c^ ± 0.12
Fraction 11	6.89–7.28	18.83 ^b^ ± 0.07	25.34 ^b^ ± 0.09
Fraction 12	7.28–7.67	45.42 ^e^ ± 0.06	32.15 ^d^ ± 0.16
Fraction 13	7.67–8.06	12.31 ^a^ ± 0.08	21.69 ^a^ ± 0.13
Fraction 14	8.06–8.44	57.33 ^h^ ± 0.21	40.23 ^g^ ± 0.19
Fraction 15	8.44–8.83	43.21 ^d^ ± 0.11	32.73 ^e^ ± 0.10
Fraction 16	8.83–9.22	49.80 ^f^ ± 0.17	34.89 ^f^ ± 0.08
Fraction 17	9.22–9.61	NA	NA
Fraction 18	9.61–10.00	NA	NA

Note: The free radical scavenging activity expressed an average value of IC_50_ ± S.D. (n = 3); the values with different lowercase letters in the same column indicate significant differences (*p*-value ≤ 0.05) compared to the control, and NA is not observed.

**Table 2:** The peptide information and accession number of matched proteins.

Peptide ID.	Peptide Sequence	Molecular Weight (Da)	ALC Score (%)	Accession Number of Protein Matching
1	PEHYLDHFKL	1297.65	98	XP_015644246.1
2	FYDPKTPFF	1160.55	97	XP_015644246.1
3	VPAGVAHW	835.43	97	XP_015627451.1
4	LKELGDKVPAPVKE	1521.88	96	XP_015619775.1
5	LDDPAKKLVFGGSA	1416.76	96	AAD10374.1
6	PASVAHW	766.38	96	EEE55040.1
7	LDDPAKKLVF	1144.65	96	AAD10374.1
8	AKLPPGSD	783.41	96	BAA28632.1
9	LLKLPTL	796.54	96	Not match found.
10	TLKYPLE	862.48	96	Not match found.
11	DVVHSHASN	964.44	96	AFN27052.1

## Data Availability

The original contributions presented in the study are included in the article/Supplementary Materials, further inquiries can be directed to the corresponding author.

## Supplementary Materials

The following supporting information can be downloaded at https://www.mdpi.com/article/10.3390/foods13152467/s1, Supplementary data: The daughter mass spectrum shows y-ion and b-ion series used for construction peptide sequence. The red line shows the derivation y-ion series of each spectrum and blue line shows the b-ion series of each spectrum. Illustration of daughter mass spectrum of (A) PEHYLDHFKL, (B) FYDPKTPFF, (C) VPAGVAHW, (D) LKELGDKVPAPVKE, (E) LDDPAKKLVFGGSA, (F) PASVAHW, (G) LDDPAKKLVF, (H) AKLPPGSD, (I) LLKLPTL, (J) TLKYPLE, and (K) DVVHSHASN. Figure S1: Cell viability of L929 cells treated with octapeptide #3 at concentrations ranging from 7.8125 to 1000 µg/mL. Error bars represent the standard deviation from three biological replicates. The x-axis represents the concentration of octapeptide #3 (µg/mL), and the y-axis represents the percentage of cell viability.
