# Supplementary material for: Protective Effects of an Octapeptide Identified from Riceberry™ (Oryza sativa) Protein Hydrolysate on Oxidative and Endoplasmic Reticulum (ER) Stress in L929 Cells
Source: Foods. 2024 Aug 5;13(15):2467. doi: 10.3390/foods13152467 (PMC11312331; doi:10.3390/foods13152467)
Supplement: Supplementary file 1 [file foods-13-02467-s001.zip › foods-3096733-supplementary.pdf]

Supplementary data

Protective Effects of an Octapeptide Identified from Riceberry™ (*Oryza sativa*) Protein Hydrolysate on Oxidative and Endoplasmic Reticulum (ER) Stress in L929 Cells

Sucheewin Krobthong [1,†], Theeranuch Jaroenchuensiri [1,†], Yodying Yingchutrakul [2], Pichayapa Sukmak [3], Wonnop Visessanguan [2], Pawin Pongkorpsakol [3], Tatpong Tulyananda [4,*] and Chanat Aonbangkhen[1,5,*]

[1] Center of Excellence in Natural Products Chemistry (CENP), Department of Chemistry, Faculty of Science, Chulalongkorn University, Bangkok 10330, Thailand; sucheewin.k@chula.ac.th (S.K.); pream.theeranuch@gmail.com (T.J.)

[2] National Center for Genetic Engineering and Biotechnology, National Science and Technology Development Agency (NSTDA), Pathum Thani 12120, Thailand; yodying.yin@biotec.or.th (Y.Y.); wonnop@biotec.or.th (W.V.)

[3] Princess Srisavangavadhana College of Medicine, Chulabhorn Royal Academy, Bangkok 10210, Thailand; pichayapasukmak@gmail.com (P.S.); pawin.pon@cra.ac.th (P.P.)

[4] Plant Biology & Astrobotany Laboratory, School of Bioinnovation and Bio-Based Product Intelligence, Faculty of Science, Mahidol University, Salaya Campus, Nakhon Pathom 73170, Thailand

[5] Center of Excellence on Petrochemical and Materials Technology, Chulalongkorn University, Bangkok 10330, Thailand

*Correspondence: tatpong.tul@mahidol.edu (T.T.); chanat.a@chula.ac.th (C.A.)

†These authors contributed equally to this work.

(A)

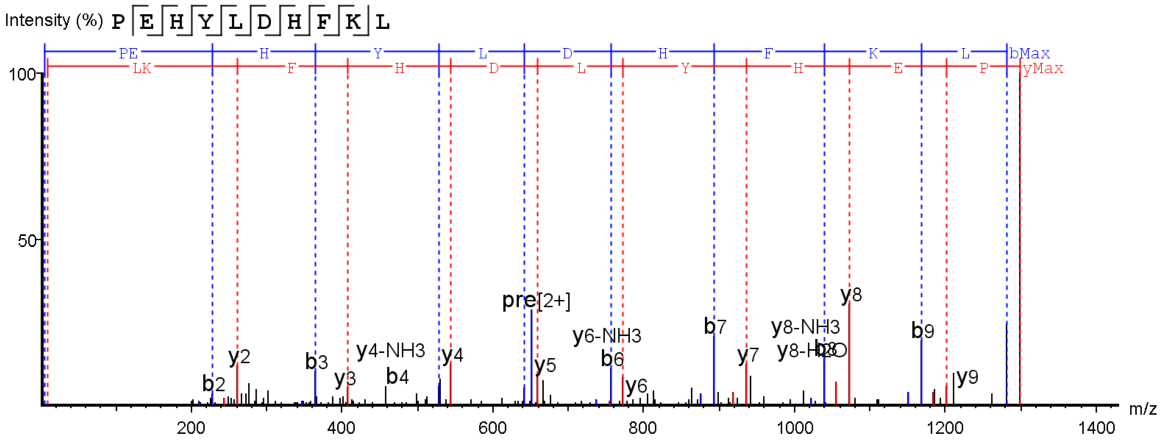

(B)

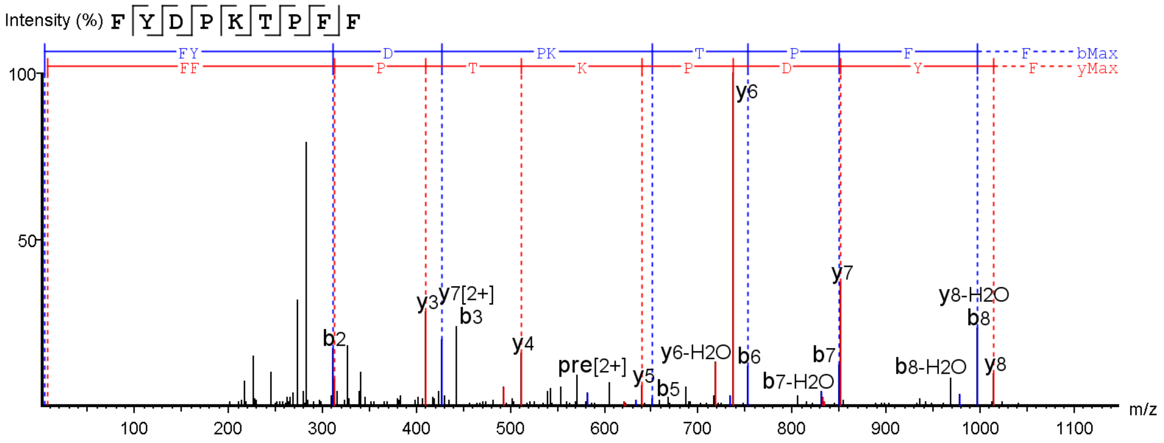

Supplementary data: The daughter mass spectrum shows y-ion and b-ion series used for construction peptide sequence. The red line shows the derivation y-ion series of each spectrum and blue line shows the b-ion series of each spectrum. Illustration of daughter mass spectrum of (A) PEHYLDHFKL, (B) FYDPKTPFF, (C) VPAGVAHW, (D) LKELGDKVPAPVKE, (E) LDDPAKKLVFGGSA, (F) PASVAHW, (G) LDDPAKKLVF, (H) AKLPPGSD, (I) LLKLPTL, (J) TLKYPLE, and (K) DVVHSHASN.

(C)

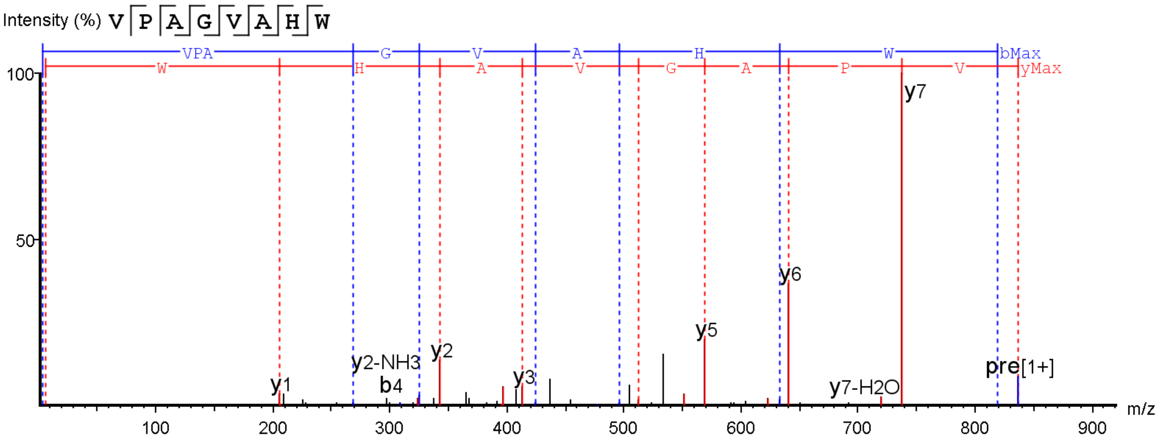

(D)

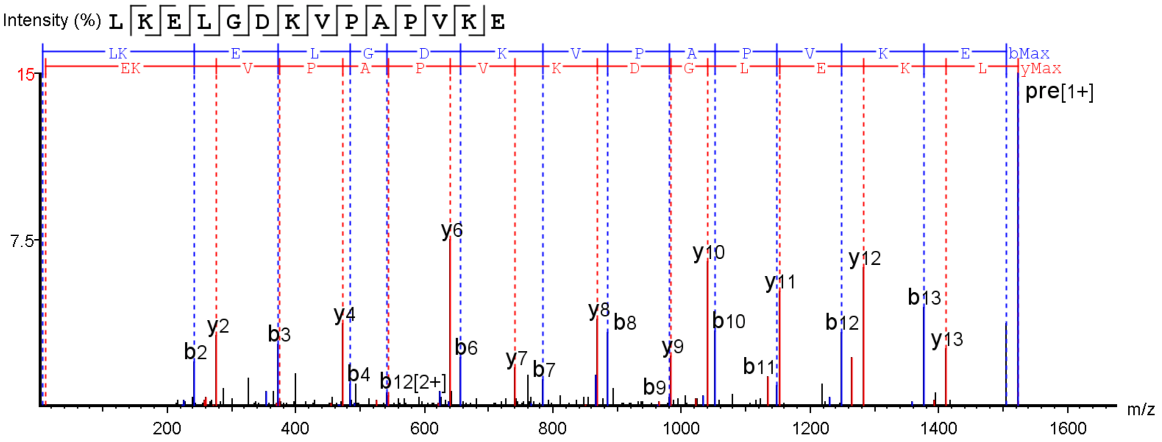

Supplementary data (cont.): The daughter mass spectrum shows y-ion and b-ion series used for construction peptide sequence. The red line shows the derivation y-ion series of each spectrum and blue line shows the b-ion series of each spectrum. Illustration of daughter mass spectrum of (A) PEHYLDHFKL, (B) FYDPKTPFF, (C) VPAGVAHW, (D) LKELGDKVPAPVKE, (E) LDDPAKKLVFGGSA, (F) PASVAHW, (G) LDDPAKKLVF, (H) AKLPPGSD, (I) LLKLPTL, (J) TLKYPLE, and (K) DVVHSHASN.

(E)

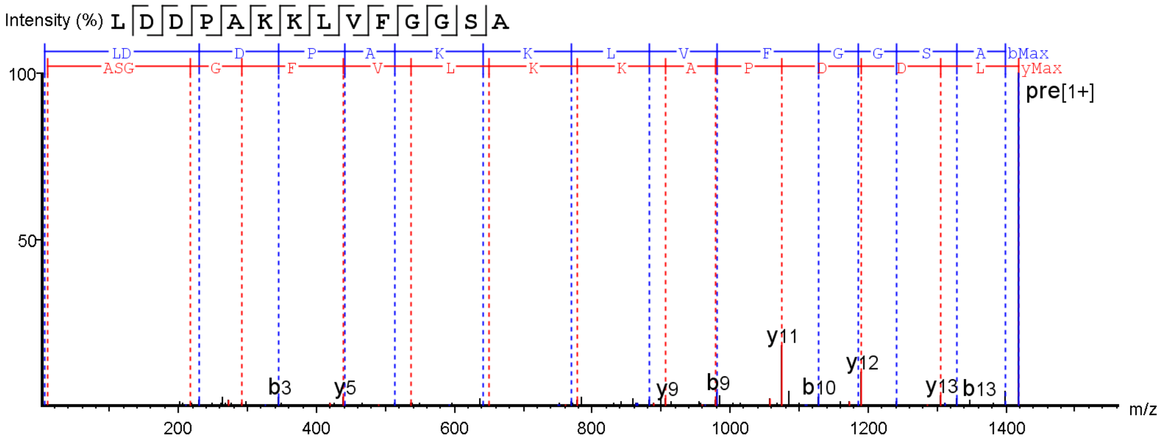

(F)

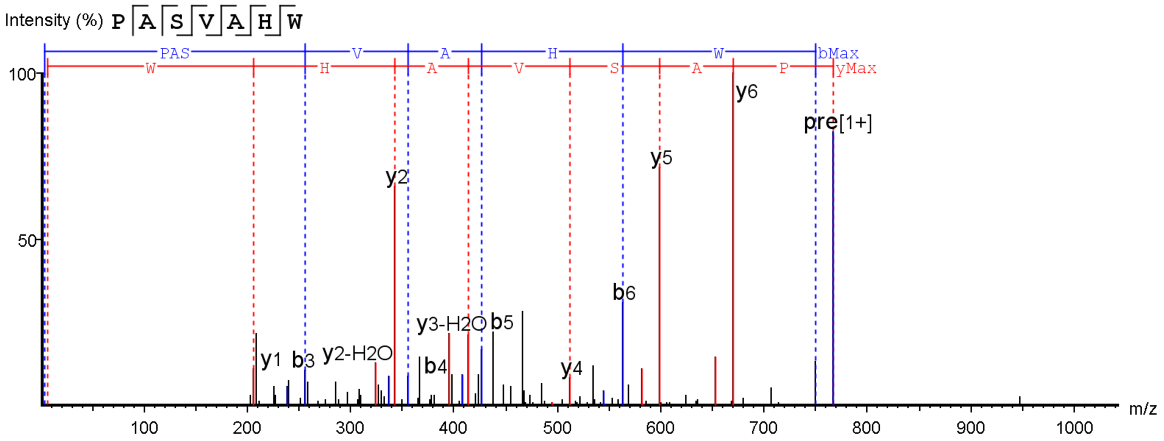

Supplementary data (cont.): The daughter mass spectrum shows y-ion and b-ion series used for construction peptide sequence. The red line shows the derivation y-ion series of each spectrum and blue line shows the b-ion series of each spectrum. Illustration of daughter mass spectrum of (A) PEHYLDHFKL, (B) FYDPKTPFF, (C) VPAGVAHW, (D) LKELGDKVPAPVKE, (E) LDDPAKKLVFGGSA, (F) PASVAHW, (G) LDDPAKKLVF, (H) AKLPPGSD, (I) LLKLPTL, (J) TLKYPLE, and (K) DVVHSHASN.

(G)

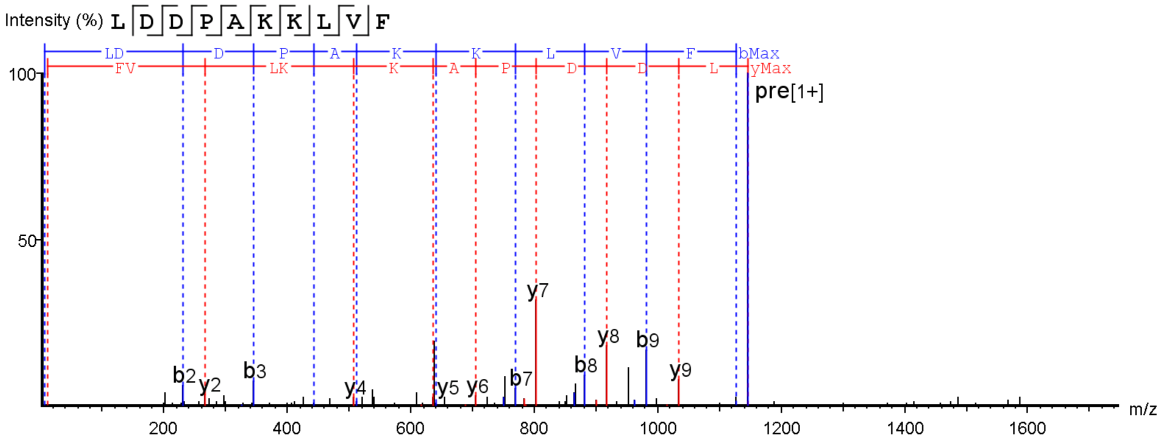

(H)

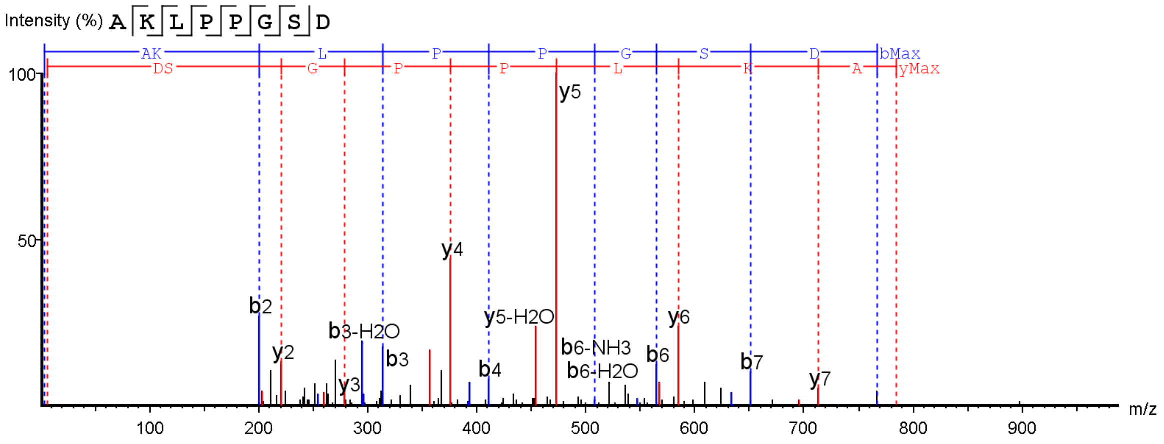

Supplementary data (cont.): The daughter mass spectrum shows y-ion and b-ion series used for construction peptide sequence. The red line shows the derivation y-ion series of each spectrum and blue line shows the b-ion series of each spectrum. Illustration of daughter mass spectrum of (A) PEHYLDHFKL, (B) FYDPKTPFF, (C) VPAGVAHW, (D) LKELGDKVPAPVKE, (E) LDDPAKKLVFGGSA, (F) PASVAHW, (G) LDDPAKKLVF, (H) AKLPPGSD, (I) LLKLPTL, (J) TLKYPLE, and (K) DVVHSHASN.

(I)

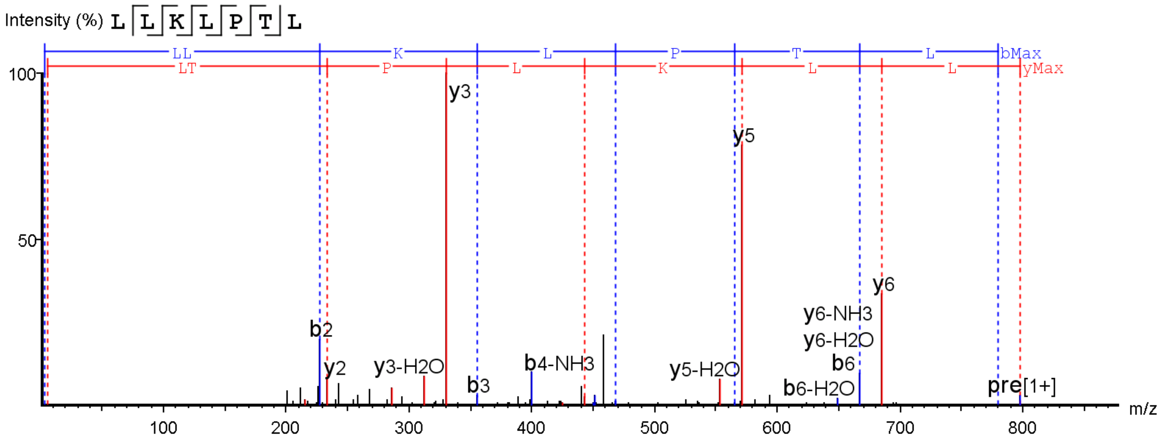

(J)

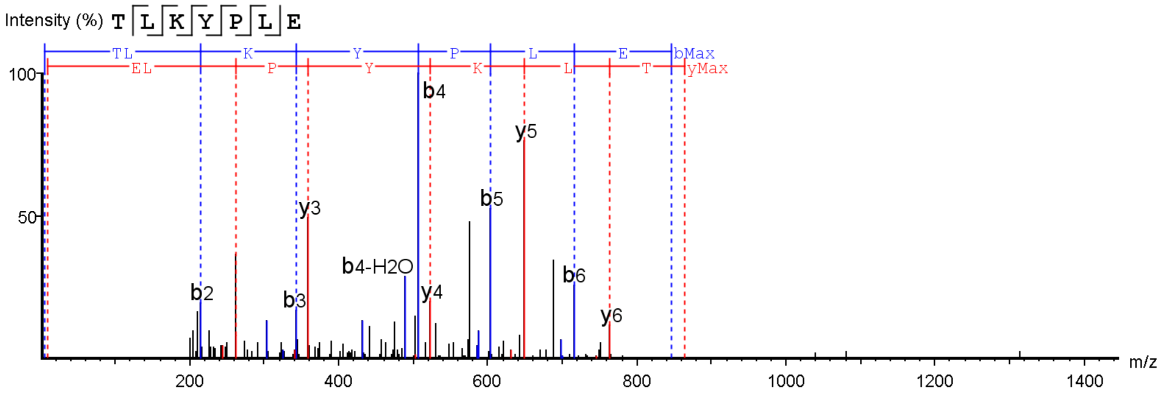

Supplementary data (cont.): The daughter mass spectrum shows y-ion and b-ion series used for construction peptide sequence. The red line shows the derivation y-ion series of each spectrum and blue line shows the b-ion series of each spectrum. Illustration of daughter mass spectrum of (A) PEHYLDHFKL, (B) FYDPKTPFF, (C) VPAGVAHW, (D) LKELGDKVPAPVKE, (E) LDDPAKKLVFGGSA, (F) PASVAHW, (G) LDDPAKKLVF, (H) AKLPPGSD, (I) LLKLPTL, (J) TLKYPLE, and (K) DVVHSHASN.

(K)

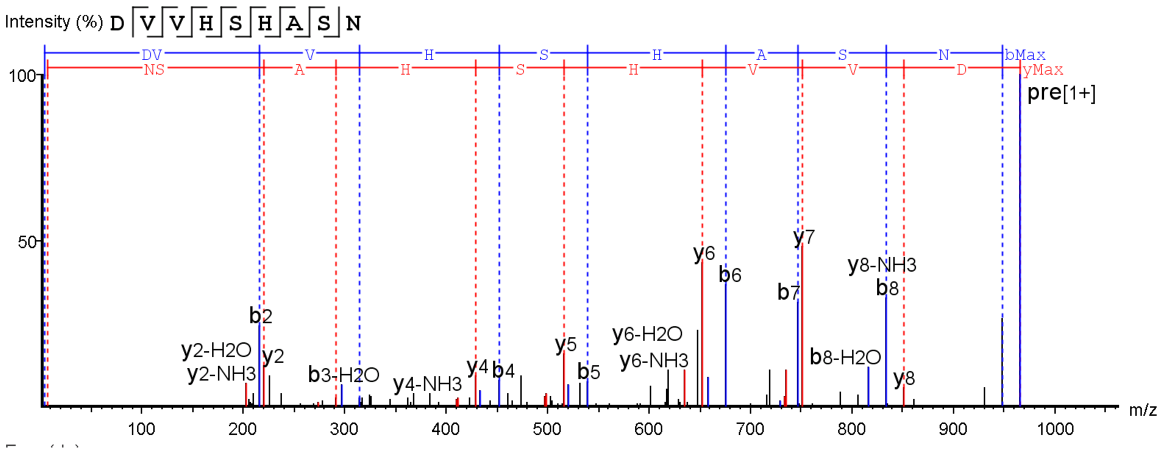

Supplementary data (cont.): The daughter mass spectrum shows y-ion and b-ion series used for construction peptide sequence. The red line shows the derivation y-ion series of each spectrum and blue line shows the b-ion series of each spectrum. Illustration of daughter mass spectrum of (A) PEHYLDHFKL, (B) FYDPKTPFF, (C) VPAGVAHW, (D) LKELGDKVPAPVKE, (E) LDDPAKKLVFGGSA, (F) PASVAHW, (G) LDDPAKKLVF, (H) AKLPPGSD, (I) LLKLPTL, (J) TLKYPLE, and (K) DVVHSHASN.

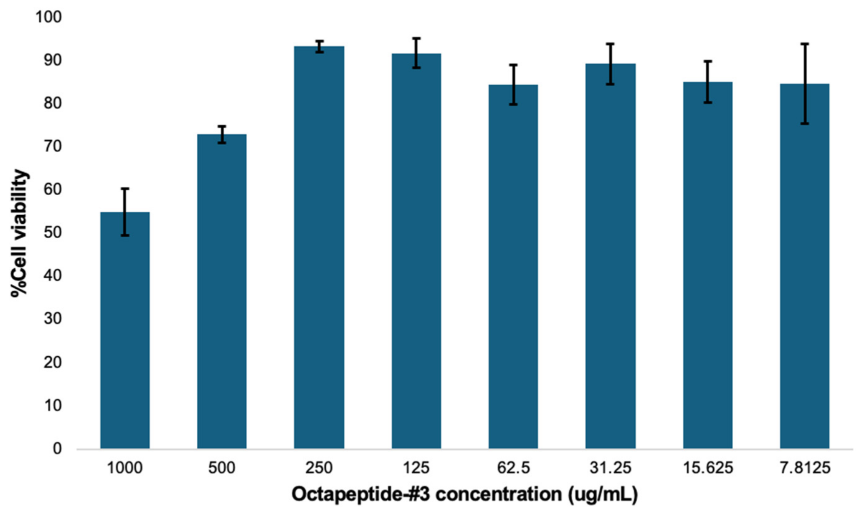

Supplementary Figure S1: Cell viability of L929 cells treated with octapeptide #3 at concentrations ranging from 7.8125 to 1000 µg/mL. Error bars represent the standard deviation from three biological replicates. The x-axis represents the concentration of octapeptide #3 (µg/mL), and the y-axis represents the percentage of cell viability
